# Molecular Biomarkers in Sinonasal Cancers: New Frontiers in Diagnosis and Treatment

**DOI:** 10.1007/s11912-021-01154-3

**Published:** 2022-01-20

**Authors:** Mario Turri-Zanoni, Giacomo Gravante, Paolo Castelnuovo

**Affiliations:** 1 Division of Otorhinolaryngology, Department of Biotechnology and Life Sciences, University of Insubria, Ospedale di Circolo e Fondazione Macchi, Via Guicciardini 9, 21100 Varese, Italy; 2 Head and Neck Surgery & Forensic Dissection Research Center (HNS&FDRc), Department of Biotechnology and Life Sciences, University of Insubria, Varese, Italy

**Keywords:** Biomarkers, Immunotherapy, Intestinal-type adenocarcinoma (ITAC), INI-1, Olfactory neuroblastoma, Mucosal melanoma, Neuroendocrine carcinoma (SNEC), Paranasal sinus cancer, PD-L1, Sinonasal undifferentiated carcinoma (SNUC), Targeted therapies

## Abstract

**Purpose of Review:**

Sinonasal tumors are rare and heterogeneous diseases which pose challenges in diagnosis and treatment. Despite significant progress made in surgical, oncological, and radiotherapy fields, their prognosis still remains poor. Therefore, alternative strategies should be studied in order to refine diagnosis and improve patient care.

**Recent Findings:**

In recent years, in-depth molecular studies have identified new biological markers, such as genetic abnormalities and epigenetic variations, which have allowed to refine diagnosis and predict prognosis. As a consequence, new histological entities have been described and specific subgroup stratifications within the well-known histotypes have been made possible. These discoveries have expanded indications for immunotherapy and targeted therapies in order to reduce tumor spread, thus representing a valuable implementation of standard treatments.

**Summary:**

Recent findings in molecular biology have paved the way for better understanding and managing such rare and aggressive tumors. Although further efforts need to be made in this direction, expectations are promising.

## Introduction

Sinonasal tumors are rare and account for 3 to 5% of malignancies of the head and neck (H&N), and 0.2 to 0.8% of all tumors [1]. Diagnosis is often late and in the advanced stage due to tumor slow growth and non-specificity of the symptoms which patients often neglect [2]. The average age of presentation is between 50 and 60 years with a higher prevalence in males [3]. A variety of histological cancer subtypes may arise in this region with different natural histories. Occupational hazards, genetic mutations, viral infections can be considered etiological agents in several tumors [4]. Most frequent malignancies of the sinonasal tract originate from the surface epithelium, in particular squamous cell carcinoma (SCC) and non-salivary-type adenocarcinoma (nsADC). The first is the most common tumor in the USA, typically originating from the maxillary sinus (60%); the latter is the most common in Europe, arising in the ethmoid sinuses (85%) and olfactory region (13%) [1]. However, the sinonasal tract can be affected by a wide range of cancers, which differ markedly from each other in their clinical behavior. Despite recent advances in diagnosis and treatment, including minimally invasive endoscopic resection, neoadjuvant chemotherapy, and particle radiotherapy, the prognosis still remains dismal with a high recurrence rate and poor survival outcomes. For this reason, studies predominantly focused on the molecular fingerprint of each specific histotype, are paramount to engage new promising treatment strategies. Moreover, recent molecular studies enabled the discovery of new rare entities that require further efforts for understanding their natural history and choosing the appropriate therapeutic strategy.

## Squamous Cell Carcinoma

SCC is a malignant epithelial tumor arising from the epithelium lining the nasal cavity and paranasal sinuses. The histologic subtypes of this tumor include keratinizing SCC (KSCC, 70% of cases, graded into well, moderately, and poorly differentiated), non-keratinizing SCC (NKSCC, 20% of cases), and other less frequent variants, such as spindle cell SCC (10% of cases) [5]. Exposure to chemical substances such as polycyclic aromatic hydrocarbons, glues, formaldehyde, chrome, nickel, and various compounds used in the textile industry has been associated with sinonasal SCC cancerogenesis [6, 7]. Chronic inflammation, supported by irritating substances, may have a role in converting normal respiratory epithelium into a squamous metaplasia and subsequent dysplasia, outlining the subsequent conversion in the so-called de novo SCC, associated with worse prognosis [4, 7]. In addition, precursor lesions are represented by Schneiderian papillomas (exophytic, inverted, and oncocytic type), with potential of cancerization ranging from 2 to 27% [7]. The role of the human papilloma virus (HPV) in sinonasal cancer is still debated [7]. HPV infection was identified in 16–19% of KSCC and more consistently in NKSCC (50%), with types 16 and 18 as the most common. In this context, NKSCC appears to be a distinct histopathologic and molecular disease from the keratinizing one. The first is characterized by high prevalence of high-risk HPV DNA, overexpression of p16 protein, high Ki-67 labeling index, and negative or low p53 reactivity; the latter is a tumor more frequently related to cigarette smoking, p53 anomalies, and low prevalence of HPV positivity. The presence of HPV is associated with a favorable outcome whereas TP53 mutation, detected in 30–75%, is associated with worse prognosis [6]. Among NKSCC, a novel subtype with DEK-AFF2 fusion was recently reported, which showed an encouraging preliminary response to immune checkpoint inhibitors [8], and needs further efforts to investigate the mechanisms of that.

An association of the Epstein-Barr virus (EBV) and a high risk of metastatic and lymph node spread in sinonasal SCC has been found [9], but such findings should be further investigated. Epidermal growth factor receptor (EGFR) mutations, frequently observed in inverted papilloma (IP) and associated with low risk of SCC transformation [7], could represent a potential target in the prognosis and treatment of this cancer type [10]. In this regard, EGFR protein expression was associated with significantly shorter overall and disease-free survival in a series of 70 sinonasal SCC [11]. Moreover, Udager et al. found that the irreversible EGFR inhibitor, neratinib, strongly inhibited EGFR signaling and its downstream molecules Mek and Akt in a sinonasal SCC cell line [12]. Similarly, amplification of fibroblast growth factor receptors 1 (FGFR1), found in 20% of SCC cases, represents a potential molecular target with FGFR-inhibitors [4, 10]. Moreover, activation of PI3K/Akt/mTOR pathway through PTEN loss and overexpression of Akt and mTOR observed in SCCs has been described as a potential option for targeted therapies in a preclinical setting [13••]. Finally, some tumoral cells may express the programmed death ligand-1 (PD-L1), which blocks the interaction between the programmed death-1 (PD-1) receptor and T lymphocytes, inhibiting their activation and suppressing the immune response. Although PD-L1 expression seems not to be directly correlated with prognosis, it can be used to select patients who may benefit from therapy with immune checkpoint inhibitors [14].

The potential prognostic role of selected biomarkers was also investigated in several studies. If on one side the P53 status plays a controversial role as prognosticator [15], on the other hand, the overexpression of TrkB and pS6 seems to be associated with more advanced grade and stage, with worse survival rates [13••, 16].

## Intestinal-Type Adenocarcinoma

Intestinal-type adenocarcinoma (ITAC) is the most common nsADC and occurs predominantly in the ethmoid sinuses (40–85%) [5, 17]. Occupational exposure represents a key point in ITAC cancerogenesis, demonstrated in about 88% of cases [18]. The most important risk factor is exposure to wood dust, followed by products in the textile industry, leather dust, and formaldehyde; debated is the role nickel/chromium compounds and asbestos [6, 19].

ITACs consist of the proliferation of dysplastic columnar cells with interspersed goblet cells, forming papillae and glands. Paneth cells and endocrine cells are also present in varying proportions. The spectrum of differentiation covers well-differentiated to poorly-differentiated tumors, stratified as papillary, colonic, and solid, or mixed, according to the Barnes classification [20]. A low percentage of cases shows abundant mucus, resulting in two different growth patterns: mucinous type and signet ring type [20].

Similarly, Kleinsasser and Schroeder divided ITAC into four subtypes: papillary-tubular cylinder cell, later graded from I to III, alveolar goblet, signet ring cell, and transitional, with the signet ring variant associated with the worst prognosis [21]. The differential diagnosis between ITAC and non-intestinal-type adenocarcinomas (n-ITAC) is made on morphologic grounds, supported by immunohistochemistry [19]. Metastatic gastrointestinal tumors, sharing many common features with ITACs, must be excluded by clinical and/or imaging findings [22]. The genetic alterations in sinonasal ITAC partially overlap with those of colorectal adenocarcinoma. TP53 is the most frequently mutated gene (40–50%), while APC, KRAS, and BRAF mutations are present in a minor subset. EGFR overexpression and gene amplification have been found in a subgroup; reports of overexpression of MET and nuclear ß-catenin expression were also described [23•]. A recent study based on next-generation sequencing (NGS) has identified recurrent somatic sequence variants in PIK3CA, APC, ATM, KRAS, NF1, LRP1B, BRCA1, ERBB3, CTNNB1, NOTCH2, and CDKN2A [24]. Genetic aberrations leading to loss of PTEN, CDH1, DCC, and APC often correlate with advanced stages and poor prognosis, as observed in colorectal cancers [25]. Several methylated genes were found in this cancer by Costales et al., and, in particular, TIMP3 methylation correlated with worse survival [26]. In addition, Tomasetti et al. suggested to investigate Mir126 as a circulating biomarker, which is expressed in malignant disease but not in benign lesions, in order to early detect malignant transformation and open the doors for potential therapeutic strategies [22, 27]. Recently, innovative treatments strategies based on the aforementioned molecular alterations are emerging for specific subgroups of patients. In detail, p53 protein status may be used to predict the response to chemotherapy. Induction chemotherapy according to the PFL (cisplatin, fluorouracil, and leucovorin) scheme seems to increase disease-free survival in the presence of a wild-type or a still-efficient p53 protein, even when encoded by a mutated TP53 gene, but ineffective in case of disabled p53 protein [28]. Additionally, mutational H-RAS profile, shown in 16% of ITAC, could fit into MAPK/ERK pathway inhibitors, alone or combined with inhibitors of the cyclin-dependent kinase-4/6 [19]. Since MET-activating mutation can be found in up to 64% of ITACs, MET inhibitors represent another interesting treatment option [19]. As for SCC, the PD-L1 expression has been shown in 17% of ITACs, supporting the potential for immune checkpoint inhibitors in selected cases of metastatic disease [14].

### Non-Intestinal-Type Adenocarcinoma

n-ITAC is an utmost rare malignancy of the sinonasal tract which morphologically displays neither intestinal-type nor salivary-type adenocarcinoma’s features. It is divided into low-grade and high-grade types. Degree of necrosis, mitotic activity, and cytologic atypia are the distinguishing characteristics between the two grades. As opposite to ITAC, wood dust exposure shows no significant association with n-ITAC tumorigenesis and the immunohistochemical panel demonstrates a respiratory-type profile (CK20 − , CK7 + , CDX2 − , and villin −), with CK7 consistently expressed [29, 30]. SATB2 has been recently identified as a potential diagnostic biomarker, capable of differentiating ITAC from n-ITAC with a high degree of specificity [31]. A molecular marker helpful in the diagnosis of these tumors may be also the OTX type 1 gene [32]. In line with this, Pirrone et al. demonstrated differential immunoreactivity of OTX1 and OTX2 between ITAC and n-ITAC types, where OTX1 is only absent from ITACs while OTX2 is absent from both [33]. Positivity for markers of seromucinous differentiation (DOG1, SOX10, and S-100) defines a subset of n-ITACs called sinonasal seromucinous adenocarcinomas [17]. n-ITAC includes also a rare subtype, namely the renal cell-like adenocarcinoma, which is a low-grade glandular malignancy of the paranasal sinuses. Despite its morphologic mimicry of renal cell carcinoma, it demonstrates a seromucinous phenotype and is associated with a favorable prognosis and low recurrence rates after a free-margins surgical resection [34]. ß-catenin and mismatch repair protein expression is wild type in high-grade n-ITACs [35]; while a subset of low-grade n-ITACs (with squamous morules) shows nuclear localization of ß-catenin [36]. Overexpression of p53 may be seen [37]. In two studies reported by Andreasen et al., ETV6 rearrangement with NTRK3 (2 cases) and with RET (one case) could represent a promising target for therapy and a valid tool as a diagnostic biomarker [38, 39].

## Adenoid Cystic Carcinoma

This is the most common salivary-type sinonasal tumor [40]. Its clinical behavior is deceptive since the tumor growth is slow but with a high propensity for perineural spread and bony local invasion, with extent through the skull base, cavernous sinus, and orbit; even systemic spread takes place especially to the lung, bone, and liver, while lymph node metastasis is less frequent [41•]. Three histological variants have been described: cribriform, tubular, and solid [5]. According to the Perzin/Szanto classification system, adenoid cystic carcinoma (ACC) is divided into three grades, with grade III defined by more than 30% of solid components and associated with worse outcomes [42, 43]. Focusing on molecular subtypes and based on dominant cell type, ACC may show an epithelial-dominant trait (E-ACC) or a myoepithelial-dominant trait (M-ACC), enabling the identification of novel potential therapeutic targets and biomarkers [44]. Moreover, M-ACC is correlated with better prognosis [45]. Genes EN1, DLX6, and OTX1 represent potential drivers and therapeutic targets for ACC [44]. Specific gene rearrangements have been described, in particular MYB-NFIB fusion, found in 50–60% of cases, resulting from a *t*(6,9) translocation which fuses the MYB proto-oncogene on chromosome 6q to the NFIB gene on chromosome 9p; this leads to an increased expression of the protein Myb, involved in the tumoral growth and associated with a more aggressive clinical course. Targeted therapy of transcription factors remains currently a major challenge [46, 47]. Moreover, ACC may express NOTCH1 mutations which characterize a poor-prognosis disease, with solid histological phenotype, high tendency in liver and bone spread, and potential responsiveness to Notch1 inhibitors [48]. EGFR and c-Kit genetic abnormalities have been observed in sporadic cases, even if specific therapies against these targets have not resulted in significant clinical responses [49, 50]. Other activating mutations may involve the vascular endothelial growth factor (VEGF) and fibroblast growth factor receptor 1 (FGFR1), supporting the need for further exploration of targeting inhibitors in this field [39]. Finally, matrix metalloproteinases (MMPs) seem to have a role in promoting ACC local and distant spread, due to their disruptive capacity on extracellular and pericellular components; in particular, immunoexpression of MMP-7 and MMP-25 is associated with better survival while high tumoral expression of MMP-9 and MMP-15 is associated with poorer survival, advanced stage, and regional recurrences [51].

## HPV-Related Multiphenotypic Sinonasal Carcinoma

HPV-related multiphenotypic sinonasal carcinoma (HPV-MSC) is morphologically similar to ACC, particularly to its solid variant, but not presenting MYB, MYBL1, or NFIB fusion genes [52, 53]. The salivary gland nature of this tumor is further supported by myoepithelial cells positivity for cytokeratin, S100, actin, calponin, p63, and ductal cells positivity for CD117 and CK7. Expression of SOX10 and LEF-1 is typical in these tumors [54–57]. The presence of focal squamous differentiation within the tumor is characteristic of HPV-MSC [55]. High-risk HPV genotypes infection such as type 33, the most common, but also types 35, 52, and 56, strongly support the diagnosis [53, 58]. Although HPV-MSC has a high-grade histological appearance, it behaves indolent with frequent local relapses and only rare systemic metastases, and therefore, surgery followed by irradiation represents the standard of care [53]. Innovative biological treatment options deserve future studies.

## Olfactory Neuroblastoma

Olfactory neuroblastoma (ONB) is a malignant tumor typically arising from the olfactory neuroepithelium in the upper nasal cavity. It commonly involves the ethmoid complex, anterior skull base, and orbit, locally, while regional spread to neck nodes and systemic dissemination to brain, leptomeninges, lung, and bones may occur with a frequency ranging from 10 to 15%. Cases of ONBs with a syndrome of inappropriate antidiuretic hormone secretion (SIADH) have also been described in the literature [59, 60]. Differential diagnosis is challenging, especially in differentiating ONB from other small round blue cells tumors, such as mucosal melanoma, rhabdomyosarcoma, sinonasal undifferentiated carcinoma (SNUC), NUT carcinoma, sinonasal neuroendocrine carcinoma (SNEC), Ewing sarcoma, and pituitary adenoma [61]. The Hyams grading system is widely accepted to stratify ONB cases in four grades according to an increased level of mitotic activity, nuclear polymorphism, Wright and Flexner-Wintersteiner rosettes, subverted architecture, necrosis, and decreased amount of fibrillary matrix [62]. Several studies supported the crucial role of Hyams grade in treatment planning, given its statistically significant association with overall prognosis and recurrence rates [63, 64]. Hyams grade III and IV ONB can be considered poorly differentiated cancers, sharing common biological alterations with SNUC and poorly differentiated SNEC, thus making difficult a proper differential diagnosis. In addition, such cancers share also a high chemo-radiosensitive behavior, so that they may benefit from different protocols of neoadjuvant chemotherapy, including the cisplatin/etoposide scheme, which might be used to select responders who can be treated with exclusive chemoradiation with slightly better survival rates [62, 64].

The immunohistochemical panel includes consistent and diffuse staining for neuron­specific enolase, chromogranin A, synaptophysin, CD56, and S100 typically localized in sustentacular cells. As opposed, CK-AE1/AE3, CK-8/18, and TTF-1 staining are usually negative. However, ONBs are a heterogeneous group of tumors and the expression of cytokeratin and chromogranin A and the mutational status of IDH2 as well as DNA methylation patterns may greatly aid in the precise classification of ONB subtypes with different biological behavior [65]. Although p53 positivity does not seem to correlate to survival, the Ki67 index can be variable (2–50%), and when elevated, it is associated with an increased risk of recurrence and poorer outcomes [64, 66]. Micheloni et al. described the OTX type 2 gene (OTX2) as a useful molecular marker for the diagnosis of ONB [32]. Molecular-based subtype classification has been proposed for ONBs, dividing them into neural type and basal type, with the latter characterized by worse prognosis and higher intratumoral-infiltrating lymphocytes, providing thus a rationale for the use of immune checkpoint inhibitors in this setting [67]. Moreover, ONB can express somatostatin receptor (SSTR), in particular SSTR-2 (75%) and, less often, SSTR-5 (7.5%), both showing the highest affinity with somatostatin analogs among all SSTR families. Thereby, somatostatin analogs can be used for diagnosis, especially in case of metastatic disease, using octreotide (111In-pentetreotide) SPECT/CT and, more recently, Gallium-68 (68 Ga) PET/CT with restricted time of image acquisition, better resolution, and lower radiation dose [68, 69]. Treatment protocols with somatostatin analogs are currently under intensive investigations, as well, especially in cases of recurrences not amenable for surgery and further irradiation [69]. Numerous chromosomal aberrations have been reported in the literature proving that gains are more frequent than losses and associated with advanced-stage tumors [66]. Moreover, genomic alteration in PI3K/mTOR signaling pathway and CDK-dependent cell cycle regulation may be involved in ONB pathogenesis, as well as CCND1 amplification, FGFR3 amplifications, and DMD gene deletions, all of them potentially opening new horizons for targeted therapy, which should be further investigated in future studies [70–73]. Topcagic et al. identified protein biomarkers potentially associated with response or resistance to classic chemotherapy drugs, in particular low ERCC1 (cisplatin sensitivity), high TOPO1 (irinotecan sensitivity), high TUBB3 (vincristine resistance), and high MRP1 (multidrug resistance) [74]. In addition, the authors demonstrated aberrations in the targetable Wnt/β-catenin signaling pathway as well as cell cycle master regulator TP53, which may confer sensitivity to WEE kinase inhibitors. Positivity for PD-L1 in ONB tumor cells is variously described in the literature and it might open the door for studies assessing the efficacy of immunotherapy (nivolumab, pembrolizumab) in this disease [22, 74].

## Mucosal Melanoma

Sinonasal mucosal melanoma (MM) is the most aggressive sinonasal tumor, currently characterized by early recurrence and high dissemination rates regardless of the treatment adopted. Free-margins surgery is the mainstay of treatment since it is generally considered a radio-resistant cancer [5]. A High pigmentation rate may help diagnosis, but when lacking, immunohistochemistry becomes paramount: S100 protein and SOX10 are usually strongly positive, while other melanocytic markers (HMB45, tyrosinase, melan A, MITF) have variable expression [61]. The well-known mutated genes involved in cutaneous melanoma cancerogenesis, unfortunately, have only a marginal role in MM, reporting variable frequencies of mutations, as follows: 7–30% in NRAS, 0–25% in c-KIT, 8–11% in TERT, 3–10% in BRAF (only in one study, 36%), 7% in SF3B1, and KRAS mutations reported only in anecdotic cases [23•]. Globally, the infrequent rate of BRAF V600E mutation observed in MM limits the efficacy of BRAF inhibitors, largely used in cutaneous melanoma, while the higher NRAS and c-KIT mutations rates make them potentially susceptible to NRAS and c-Kit target therapies [5, 22, 61, 75]. In this regard, sorafenib and imatinib molecules have been used in cases harboring specific mutations but without significant improvement in overall survival rates [76]. However, this lack of improvement may be explained by the fact that this treatment was generally given to those with end-stage, disseminated disease. Other potential therapeutic strategies may involve loss of PTEN and p16/INK4a, which may indicate activation of PI3K/Akt/mTOR and RAS/MAPK pathways [75]. However, genetic alterations have been found only in a small fraction of cases, thus supporting the urgent need for alternative treatment strategies. Recently, immunotherapy has shown promising results in selected cases, both in neoadjuvant and adjuvant settings, especially in terms of decreased systemic spread of disease. Hur et al. reported the use of ipilimumab, a monoclonal antibody targeting CTLA-4, in a cohort of metastatic MM, obtaining a 12.5% response rate, improved up to 23% when combined with the anti-PD1 therapy, nivolumab [77]. The concept of sequential immune checkpoint blockade with two inhibitors, such as anti-PD1 and anti-CTLA-4, merits further study to determine which patients are most likely to benefit, especially due to the potential escape oncogenic mechanisms intrinsic to MM biologic nature. MM is able to switch its oncogenic driver during a targeted therapy, in order to survive and continue its tumoral progression. Drug sensitivity may be regained upon treatment discontinuation but a permanent resistance to therapy is often detected. Thereby, longitudinal molecular studies and further deciphering in the molecular and immunological frame of MM are currently ongoing worldwide in order to better understand cellular plasticity and drive the treatment strategies [78].

## Sinonasal Undifferentiated Carcinoma

SNUC is a highly aggressive carcinoma lacking glandular or squamous features with great local aggressivity and tendency to metastasize [1]. In general, SNUC presents high chemosensitivity to cisplatin-based regimen and the partial or complete response to induction chemotherapy (IC) is considered a favorable prognostic factor, guiding the treatment choice toward definitive chemoradiation [79, 80]. Conversely, in patients who do not achieve a favorable response to IC, surgery, when feasible, seems to provide a better chance of disease control and improved survival [79].

SNUC is generally regarded as a diagnosis of exclusion given the complexities in its definition and misdiagnosis is frequent. The immunohistochemical panel stains positively for epithelial markers (AE1/AE3, CK7, CAM5.2, EMA), p16, CD117, and focal p63 and negatively for CK5/6, p40, CEA, EBER, CD34, desmin, S100 protein, and calretinin. Neuroendocrine markers (NSE, synaptophysin, chromogranin, CD56) may be present [61, 81]. CLCA2 is shown to have reduced expression in SNUC compared with SCC, leading to improved proliferation, migration, invasion, and epithelial-mesenchymal transition. Thus, restoration of CLCA2 function might be a promising therapy in the future [22, 82–84]. Recently, a SNUC subtype characterized by mutations of the metabolic enzymes IDH2 involved in the Krebs cycle has been described [85]. Jo et al. found IDH2 R172X mutations in 55% of SNUCs [86]; other mutations in the same codon (R172S, R172T, and R172M) have been described, with frequency up to 82% of cases [87]. Thus, patients presenting with IDH2 mutations may benefit from therapy with targeted IDH inhibitors enasidenib and ivosidenib, recently approved by FDA [87]. Since the mutation almost always occurs at the same codon, Dogan et al. have determined the utility of the 11C8B1 monoclonal antibody as a surrogate marker for this mutation in order to easily identify IDH2-mutant SNUC [88]. Remarkably, Dogan et al. found an improved disease-free survival and reduced lung metastasis in IDH-mutant SNUC. Interestingly, IDH2 mutations may be present even in large-cell neuroendocrine carcinomas, sharing with IDH2-mutant SNUC also similar cancer signaling pathways. Moreover, IDH2-mutated carcinomas seem to be characterized by hypermethylation and upregulation of the repressive H3K27 epigenetic mark, opening the door for a DNA methylation-based classification of SNUC [88].

## SWI/SNF Complex-Deficient Sinonasal Carcinoma

The loss of SMARCB1 (INI1) protein expression, which is a member of the chromatin-remodeling SWI/SNF complex located at 22q11.2, can be identified in selected cases of poorly or undifferentiated sinonasal epithelial cancers, referred to as SMARCB1-deficient sinonasal carcinomas. This loss represents a negative prognostic factor, associated with a high propensity for systemic spread and reduced overall survival, so that some authors proposed to define such cancers as distinct entities in their own right [85, 89]. Histologically, undifferentiated basaloid morphology, often with rhabdoid or plasmacytoid features, is recognizable. The immunohistochemical profile displays the expression of pancytokeratin (AE1/AE3), CK5, p63, CK7, and neuroendocrine markers [85, 87]. Coexisting genetic alterations reported by Dogan et al. included loss of NF2 and CHEK2 (50%), chromosome 7 gain (25%), and TP53 V157F, CDKN2A W110, and CTNNB1 S45F mutations [90]. The DNA methylation analysis described by Laco et al. found a significantly higher methylation level of the RASSF1 gene [91]. A novel entity is represented by SMARCB1-deficient sinonasal adenocarcinoma, with only few cases described in the literature so far [92]. It displays predominantly an oncocytoid/plasmacytoid cell pattern with prominent gland formation; patterns of yolk sac tumors are seen up in one-quarter of cases and may rarely be the predominant pattern. It may be misdiagnoses with high-grade intestinal or non-intestinal adenocarcinoma, myoepithelial carcinoma, or even yolk sac tumor or metastatic hepatocellular carcinoma [92].

SMARCA4 (BRG1)-deficient sinonasal carcinoma represents another uncommon tumor subtype where the SWI/SNF chromatin-remodeling complex is aberrated. Very few cases have been described in the literature so far, which were morphologically and clinically similar to INI1-deficient sinonasal cancers. As opposed to SMARCB1-deficient tumor, it shares more overlapping features with SNUC and poorly differentiated neuroendocrine carcinomas [85, 87]. Loss of additional SWI/SNF subunits, mainly the SMARCA2, is utmost rare and reported in a few cases [93, 94].

The identification of SWI/SNF complex subunits as key players in sinonasal cancer prompts intrigue in the potential efficacy of novel therapies. Targeted therapies with enhancer of zeste homolog 2 (EZH2) and cyclin-dependent kinase 4/6 (CDK4/6) inhibitors may emerge as potential options for treating tumors with SMARCA4 inactivation [95, 96]. In addition, a recent study demonstrated the susceptibility of SMARC4A-deficient ovarian and lung cancer models to bromodomain inhibitors [97]. Whether this applies in sinonasal carcinoma warrants investigation.

## NUT Carcinoma

NUT carcinoma is a highly aggressive carcinoma defined by *t*(15;19) translocation, supported by the fusion of the NUT (NUTM1) gene on chromosome 15q14 with BRD4 on chromosome 19p13. Rarely, NUT-BRD3 and NUT-NSD3 are involved in this translocation [61, 87]. Patients with non-BRD4-NUTM1 fusions (BRD3- or NSD3-NUTM1, median overall survival, 36.5 months) have significantly better survival than those with BRD4-NUTM1 fusions (median overall survival, 10 months), independently of metastatic disease extent at presentation [98]. Morphologically, it contains differentiated/undifferentiated cells with variable necrosis and numerous mitotic figures. More than 50% of nuclear labeling with a monoclonal antibody targeting NUT mutation is required for diagnosis. Bromodomain (NUT) inhibitors might represent a promising targeted treatment for such tumors presenting with a very poor prognosis to date [61, 87].

## Teratocarcinosarcoma

Teratocarcinosarcoma (TCS) is a high-grade sinonasal malignancy defined histologically by features of malignant teratoma, carcinoma, and sarcoma with fetal-like clear cell appearance, and a variety of mesenchymal elements [95]. Most likely histogenesis theory is from somatic pluripotent stem cells in the olfactory membrane [95]. Vranic et al. reported a case with trisomy 12p, a well-known cytogenetic abnormality occurring in the majority of malignant germ cell tumors [99]. NGS reported activating CTNNB1 mutation in a single case of TCS [100]. Rooper et al., interestingly, found a loss of SMARCA4 expression in 18 cases of TCS (82%) and variable positivity for Claudin-4 [95]. These results provide important information about the emerging role of SMARCA4 in sinonasal cancers and particularly suggest that TCS is on a spectrum with SMARCA4-deficient sinonasal carcinomas and could benefit from similar novel targeted therapies [95]. Moreover, the potential involvement of Wnt/β-catenin and PI3K/AKT/mTOR pathways could support specific treatments for this tumor [100, 101].

## Sinonasal Neuroendocrine Carcinoma

SNEC is a rare poorly differentiated carcinoma with neuroendocrine differentiation, characterized by a dismal prognosis and a high tendency to produce systemic metastasis [61]. It can be divided into small- and large-cell carcinomas, with different biological profiles and clinical courses [102••]. The immunohistochemical profile includes positivity for pancytokeratin (AE1/AE3), CK8/18, neuroendocrine markers (synaptophysin, chromogranin, NSE, CD56; at least one). The Ki67 mitotic index is usually more than 20%. Rooper et al. studied the status of insulinoma-associated protein 1 (INSM1) in H&N neuroendocrine carcinomas, finding a consistent positivity in all cases, thus supporting the role of INSM1 as a diagnostic biomarker for SNECs [103]. SNEC may benefit from induction chemotherapy followed by concurrent chemoradiation; surgery can be performed in non-responsive cases or as a salvage treatment [61, 64]. From a molecular viewpoint, Kovarikova et al. found an MiR-21 upregulation in several cancers, including SNEC, which seems to be associated with poor prognosis; however, further investigations are required in this direction [104]. Dogan et al., using the hierarchical clustering, described a cluster of IDH2-mutated carcinomas including not only SNUC but even large-cell SNEC, which shared a largely similar epigenetic signature. On the other hand, small-cell SNECs and SMARCB1-deficient sinonasal carcinomas seem to be molecularly distinct from IDH2-mutated carcinomas, as supported by the distribution of ARID1A mutations, which were common in small-cell SNEC but not among IDH2-mutant cancers [88]. Lastly, based on the presence of neuroendocrine and nonneuroendocrine tumoral components, a new entity has been recently described, namely the mixed neuroendocrine-nonneuroendocrine neoplasm (MiNEN) [64]. It is characterized by the presence of at least 30% of the neuroendocrine component within a nonneuroendocrine cancer (e.g., ITAC, SCC), and unfortunately, it bears a very poor prognosis [64, 105]. Due to the extreme rarity of this disease, no studies are available concerning potential biomarkers for improved diagnosis, prognosis, and treatment.

### Chondrosarcoma

Chondrosarcoma of the sinonasal tract is an uncommon tumor arising from hyaline cartilage. They are divided into low, intermediate, and high grade, with the former showing indolent/slow growth [5]. Mesenchymal chondrosarcoma represents an aggressive high-grade variant with a biphasic component of undifferentiated small blue round cells with islands of well-differentiated hyaline cartilage. The paucity of cartilage may result in frequent misdiagnosis. The immunohistochemical profile includes positivity to CD99, CD56, NSE, GFAP, desmin, and synaptophysin, whereas negativity to keratins and S100 protein. SOX9, a regulator of chondrogenesis, is the most helpful marker because it is non-reactive in the other small round blue cell tumors [61, 106]. Moreover, HEY1-NCOA2 fusions are held by 80% of tumors, providing another valid diagnostic tool [107]. IDH1/2 mutations were associated with longer disease-free survival and open to therapeutic targeted strategies with IDH-inhibitor therapy [90].

## Undifferentiated Small Round Cell Sarcomas

Ewing Sarcoma (ES) is a primitive small round cell sarcoma defined by fusions involving members of the FET (predominantly *EWSR1* or *FUS*) and ETS (most commonly including *FLI1*, *ERG*, *ETV1*, *ETV4*, or *FEV*) gene families. The translocation of the EWSR1 gene, located on 22q12, and the FLI‐1 gene, located on 11q24, resulting in a *t*(22;11), is the most frequently observed. Translocation variants are detected in 10–15% of cases [61, 108]. It occurs mainly in young male patients and is classified in two entities: skeletal form, typically occurring in the long bones of the extremities, and extra-skeletal form, such as those involving paranasal sinuses [109]. Immunohistochemical positivity to neural markers (NSE, S100, synaptophysin, chromogranin) is often reported. A combination of CD99, strongly expressed, and NKX2.2, found in around 95% of ES, may improve the diagnosis [110, 111]. Multidisciplinary treatment is required, but local recurrences and distant metastases are usually soon developed, within 2 years after the initial presentation. Moreover, p53 mutation has been reported as a poor prognosticator [61]. Adamantinoma-like Ewing sarcoma (ALES) is a rare variant of ES defined by complex epithelial differentiation, with an expression of pancytokeratin, CD99, p40, and synaptophysin and frequent keratin pearl formation [112].

Rhabdomyosarcoma with TFCP2 rearrangement (TFCP2-RMS) is a high-grade rhabdomyosarcoma, characterized by a fusion of TFCP2 to EWSR-1 or FUS. Most TFCP2-RMS arise in bone, less frequently in soft tissue. There is a striking predilection for craniofacial bones, in decreasing order of frequency the mandible, maxilla, and skull bones, from where TFCP2-RMS commonly infiltrates into the soft tissues of the mouth, nose, and neck. They are associated with a poor prognosis, including regional and distant spread, with high rates of disease-related death. Their association with ALK overexpression might represent a therapeutic target [113, 114].

## Biphenotypic Sinonasal Sarcoma

Biphenotypic sinonasal sarcoma (BSNS) is a low-grade sinonasal sarcoma with neural and myogenic differentiation. It demonstrates a slowly progressive growth and encouraging overall survival outcomes, with possible local recurrences reported in a percentage up to 32% of cases [115–117]. Histologically, BSNS is characterized by “herringbone” fascicular pattern, “staghorn” vessels, and consistent immunohistochemical positivity for S100, smooth muscle actin (SMA), calponin, and b-catenin. Moreover, it can also show a variable expression of desmin, myogenin, and factor XIIIa, while it is negative for cytokeratin and SOX10 [118]. Crucial for diagnosis is the chromosomal translocation *t*(2;4)(q35;q31.1), resulting in a PAX3-MAML3 fusion protein, which is a potent transcriptional activator of PAX3 response elements [119]. Alternative PAX3 partners include FOXO1, NCOA1, NCOA2, and WWTR1 [117].

## Glomangiopericytoma

Glomangiopericytoma **(**GPC), also named sinonasal hemangiopericytoma, is a rare mesenchymal tumor unique to the sinonasal tract and characterized by prominent perivascular growth. GPC shows epithelioid cells in a perivascular pattern with frequent perivascular hyalinization. It stains positively for cytoplasmic SMA, Vimentin, and nuclear β-catenin in 80–100% cases [120]. GPC expresses lymphoid enhancer-binding factor 1 (LEF1), a protein downstream from β-catenin. Moreover, mutation analysis displays CTNNB1 exon 3 mutations, specifically in the GSK3beta region, with the activation of the Wnt signaling pathway [121, 122].

## Conclusions

Sinonasal cancers are rare and extremely heterogeneous tumors. Given their aggressive behavior, novel diagnostic, prognostic, and therapeutic biomarkers are strongly required. Genetic and epigenetic changes described so far, as summarized in Table 1, are promising but it is still difficult to utilize all of them as biomarkers in daily clinical practice. Therefore, large multi-center studies are necessary to further validate these findings, in order to build a comprehensive model of carcinogenesis for each sinonasal cancer subtype. This will finally support the translation of personalized cancer medicine into the clinical management of sinonasal cancers, which will surely lead to improved survival for this challenging group of cancers.

**Table 1:** Summary of molecular biomarkers in sinonasal cancers

*Tumor histotype*	*Immunohistochemical panel*	*Molecular profile*
SCC	Positivity for pancytokeratins, CK5/6, p63, p40, EMA TrkB and pS6 are negative prognostic biomarkers	TP53, EGFR, FGFR, PD-L1, PI3K/Akt/mTOR, HPV-DNA, DEK-AFF2 fusion
ITAC	Positivity for pancytokeratins, CK20, CDX2, villin, MUC2 Negativity for CK7	TP53, EGFR, MET, KRAS, BRAF, PD-L1, PTEN, CDH1, DCC, APC, CTNNB1, TIMP3, PIK3CA, ATM, NF1, LRP1B, BRCA1, ERBB3, NOTCH2, CDKN2A, Mir126
n-ITAC	Positivity for pancytokeratins, CK7, CDX2, MUC2 Negativity for CK20, villin	OTX1, ETV6-NTRK3 fusion, ETV6-RET fusion
ACC	Positivity for CD117 (inner epithelial cells) and p63, SMA (peripheral myoepithelial cells). MMP-7,—25 (better prognosis) and MMP-9, -15 (poor prognosis)	MYB-NFIB fusion t(6,9), KIT, EGFR, FGFR1, VEGF, NOTCH1, EN1, DLX6, OTX1
HPV-MSC	Positivity for cytokeratin, S100, actin, calponin, p63 (myoepithelial cells) and CD117, CK7 (ductal cells). SOX10, LEF-1	Negativity for MYB, MYBL1, or NFIB fusion genes
ONB	Positivity for NSE, S100, chromogranin A, synaptophysin, CD56 Negativity for pancytokeratin, CK-8/18, TTF-1	OTX2, SSTR2 (75%), SSTR5 (7,5%), PI3K/mTOR, CCND1, FGFR3, DMD, PD-L1, methylation status
MM	Positivity for S100, SOX10, HMB45, tyrosinase, melan A, MITF	NRAS (G12 hot-sport mutation), KIT, TERT, BRAF, SF3B1, KRAS, PTEN, p16/INK4a, PD-L1
SNUC	Positivity for pancytokeratin, CK7, OSCAR, CAM5.2, EMA, p16, CD117, p63 Variable expression of neuroendocrine markers Negativity for CK5/6, p40, CEA, EBER, CD34, desmin, S100, calretinin	Particular subtype: IDH-mutant SNUC
SWI/SNF complex-deficient sinonasal carcinoma	Positivity for pancytokeratin, neuroendocrine markers	SMARCB1, SMARCA4, SMARCA2
NUT carcinoma	Positivity for pancytokeratin, CK5/6, p63, p40	NUT-BRD4 fusion t(15,19), rarely NUT-BRD3 and NUT-NSD3
Teratocarcinosarcoma	Positivity for epithelial, mesenchymal, and neuroepithelial markers Negativity for PLAP, alpha-fetoprotein, hCG, and CD30	Trisomy 12p, CTNNB1, SMARCA4, CLDN4, Wnt/β-catenin, PI3K/AKT/mTOR
SNEC	Positivity for pancytokeratin, CK8/18, neuroendocrine markers (synaptophysin, chromogranin, NSE, CD56—at least one) Negativity for S100, TTF-1	INSM1, Mir21, IDH2-mutations (large-cell SNEC), ARID1A-mutations (small-cell SNEC)
Chondrosarcoma	Positivity for CD99, CD56, NSE, GFAP, desmin, synaptophysin, SOX9 Negativity for keratins, S100	HEY1-NCOA2 fusion, IDH1/2 mutations
Ewing sarcoma	Positivity for CD99, NKX2.2, NSE, S100, synaptophysin, chromogranin	FET-ETS fusion, typically EWSR1-FLI1 fusion *t*(22;11)
TFCP2-RMS	Positivity for desmin, myogenin, myosin, myoglobin	TFCP2-EWSR-1/FUS fusion
BSNS	Positivity for S100, SMA, calponin, b-catenin Variable expression for desmin, myogenin, factor XIIIa	PAX3-MAML3 fusion *t*(2;4)
Glomangiopericytoma	Positivity for SMA, vimentin, nuclear β-catenin Negativity for pancytokeratin, Bcl-2, CD34, CD99, CD117, S100, STAT6	CTNNB1, LEF1

Abbreviations: *SCC*, squamous cell carcinoma; *ITAC*, intestinal-type adenocarcinoma; *n-ITAC*, non-intestinal-type adenocarcinoma; *ACC*, adenoid cystic carcinoma; *HPV-MSC*, HPV-related multiphenotypic sinonasal carcinoma; *ONB*, olfactory neuroblastoma; *MM*, mucosal melanoma; *SNUC*, sinonasal undifferentiated carcinoma; *SNEC*, sinonasal neuroendocrine carcinoma; *TFCP2-RMS*, rhabdomyosarcoma with TFCP2 rearrangement; *BSNS*, biphenotypic sinonasal sarcoma
